# Prognostic factors for recurrence-free and overall survival after adrenalectomy for metastatic carcinoma: a retrospective cohort pilot study

**DOI:** 10.1186/1471-2490-14-41

**Published:** 2014-05-23

**Authors:** Eu Chang Hwang, Insang Hwang, Seung Il Jung, Taek Won Kang, Dong Deuk Kwon, Suk Hee Heo, Jun Eul Hwang, Sung Gu Kang, Seok Ho Kang, Jeong Gu Lee, Je Jong Kim, Jun Cheon

**Affiliations:** 1 Department of Urology, Chonnam National University Medical School, Gwangju, Korea; 2 Department of Radiology, Chonnam National University Medical School, Gwangju, Korea; 3 Department of Hemato-oncology, Chonnam National University Medical School, Gwangju, Korea; 4 Department of Urology, Korea University School of Medicine, 73, Inchon-ro, Seongbuk-gu, Seoul, Republic of Korea

**Keywords:** Adrenalectomy, Neoplasm metastasis, Prognosis, Survival

## Abstract

**Background:**

The survival benefits of adrenalectomy (ADx) in the setting of metastatic cancer and prognostic factors for recurrence-free (RFS) and overall survival (OS) after adrenalectomy for metastatic carcinoma are still under debate. We evaluated the impact of clinicopathological variables on RFS and OS after ADx for metastatic carcinoma in patients with primary cancer.

**Methods:**

A total of 32 patients undergoing ADx for metastatic cancer between 2004 and 2012 at two tertiary medical centers. Metastases were regarded as synchronous (<6 months) or metachronous (≥6 months) depending on the interval after primary surgery. Associations of perioperative clinicopathologic variables with RFS and OS were analyzed using Cox regression models.

**Results:**

In total, 32 patients received ADx for metastatic primary tumors located in the lung (n = 11), colon (n = 4), liver (n = 5), stomach (n = 3), kidney (n = 4), pancreas (n = 2), glottis, esophagus, cervix, and ovary (n = 1 each). The overall recurrence rate after adrenalectomy was 62.5% (n = 20). By univariate analysis, C-reactive protein, inflammation-based prognosis score, and adrenalectomy for curative intent were associated with RFS and OS. Independent prognostic factors for shorter RFS were operative method (laparoscopy HR 4.68, 95% CI 1.61-13.61, p = 0.005) and inflammation-based prognostic score (HR 11.8, 95% CI 2.50-55.7, p = 0.002). For shorter OS, synchronous metastasis (HR 3.05, 95% CI 1.07-11.94, p = 0.048) and inflammation-based prognostic score (HR 6.65, 95% CI 1.25-35.23, p = 0.026) were identified as independent prognostic factors.

**Conclusions:**

Our pilot study suggests that synchronous disease and inflammation-based prognostic score are significant prognostic factors for survival and should be considered when performing ADx for metastatic diseases.

## Background

The adrenal glands are a common site of metastases from a variable primary cancer. Approximately 25% of patients with primary cancer are found at autopsy to have metastases to their adrenal glands [1]. Indeed, it has been estimated that, in patients with a history of a previous malignancy, over 50% of newly discovered adrenal lesions are metastatic [2].

Advances in oncological and surgical therapies have led to a significant increase in the life expectancy of cancer patients and have also prolonged survival of patients with isolated or multiple metastases. Although the overall prognosis for metastatic cancer in the adrenal glands is poor, the survival duration is longer in patients who undergo adrenalectomy for metastatic cancer in comparison to that in patients who undergo resection of metastases in other visceral sites, such as the liver and lung, because adrenal metastasis is often confined within the adrenal gland itself, providing more chances to achieve en bloc removal [3-6]. It seems reasonable to apply similar criteria for selecting patients for resection of adrenal metastases, including control of extra-adrenal disease, a reasonably long disease-free interval, an acceptable patient performance status, and the absence of significant comorbidity [2]. In this regard, several studies have reported survival benefits of adrenalectomy (ADx) in the setting of metastatic cancer [7,8] Nevertheless, the prognostic factors for recurrence-free (RFS) and overall survival (OS) after adrenalectomy for metastatic carcinoma are still under debate [8-11]. Therefore, we evaluated the impacts of clinicopathological variables on the RFS and OS after ADx for metastatic carcinoma in patients with primary cancer.

## Methods

### Patients

At two tertiary medical centers in Korea (Chonnam National University Hwasun Hospital and Korea University Anam Hospital), a total of 32 patients received ADx for metastasis to the adrenal gland, irrespective of the primary cancer type, between January 2004 and December 2012. Clinical indicated metastasis was evaluated using a combination of endoscopy, computed tomographic scans of the chest and abdomen, and positron emission tomography or bone scans. The criteria for inclusion in the present study of ADx for adrenal metastasis were as follows: (1) histologically confirmed primary cancer and adrenal metastasis; (2) presence of synchronous (primary diagnosis to adrenalectomy less than six months) or metachronous (primary diagnosis to adrenalectomy more than six months) adrenal metastasis. Patients with renal cell carcinoma (RCC) with synchronous adrenal metastasis (ipsilateral or contralateral) or direct invasion of a primary cancer in the adrenal gland were excluded. Intent to treat was divided into cure and palliation. Cure was defined as a curative margin-negative (R0) resection with no evidence of residual tumor at another site. Palliation was defined as a surgical resection for symptom relief or margin-positive (R1) resection with no evidence of residual tumor at another site. Data regarding patient demographics, RFS and OS were obtained by medical record review. The recommendations of the Declaration of Helsinki for biomedical research involving human subjects were followed throughout. The study protocol was reviewed by the Institutional Review Board of Chonnam National University Hwasun Hospital and Korea University Anam Hospital and they waived the need for ethical approval in both institutions.

### Measurement of serum CRP and definition of Glasgow Prognostic Score (GPS)

Routine laboratory testing of serum CRP and albumin was performed before ADx. Serum CRP was measured by latex turbidimetric immunoassay using a HITACHI 7600 analyzer (Hitachi, Tokyo, Japan). The CRP limit of detection was 0.03 mg/dl, and 1.0 mg/dl was the upper limit of the normal range. Coefficients of variation over the range of measurements were < 5%.

Each GPS was assigned as follows: patients with both elevated CRP (>1.0 mg/dl) and low albumin (<3.5 mg/dl) received a score of 2, whereas those with only one or none of these biochemical abnormalities earned scores of 1 and 0, respectively.

### Statistics

Univariate and multivariate analyses (stepwise forward procedure) were performed using Cox proportional hazard analysis to identify risk factors affecting overall survival (OS) and recurrence-free survival (RFS). OS was defined as the period from ADx to the date of death from any cause. RFS was defined as the period from ADx to the date of disease progression or death, whichever occurred first. If neither event had occurred at the time of the last record, the patient was censored at that time. The factors included in the model were age, sex, ECOG PS, operative method (open or laparoscopic), intent to treat (palliation or cure), interval of primary diagnosis to adrenalectomy (synchronous vs. metachronous), site of metastasis, site of primary tumor, previous metastasectomy, C-reactive protein, serum albumin, and GPS. Among the factors, those with p < 0.25 were selected (on univariate analysis for RFS and OS) and included in the multivariate regression analysis using Cox proportional hazards regression model, which was performed to achieve adjusted hazard ratio (HR) to determine prognostic factors for recurrence free and overall survival. A two-tailed p < 0.05 was considered significant for all analyses. The SPSS software package, version 19.0 (SPSS Inc., Chicago, IL, USA) was used for statistical analysis.

## Results

### Patient demographics

The baseline characteristics of the 32 patients are shown in Table 1. The median patient age was 63.5 years (range, 35–82 years). The median follow-up time (from adrenalectomy to death or last follow-up date) was 10.4 months (range, 0.5-74.3 months), and median time to adrenalectomy after primary cancer diagnosis was 8.8 months (range, 0–93.8 months). A total of 27 patients (84.4%) were male, and five patients (15.6%) were female. Nineteen patients (59.4%) had synchronous adrenal metastasis and 13 had metachronous adrenal metastasis. The diagnoses of primary cancer were lung cancer (n = 11), liver cancer (n = 5), kidney cancer (n = 4), colon cancer (n = 4), gastric cancer (n = 3), and glottis, esophagus, pancreas, and ovary cancer (one each). The median size of the largest metastatic adrenal tumor was 3.5 cm, with a range of 1–10 cm. In 21 patients (65.2%), CRP was elevated (>1 mg/dL), and 12 patients (37.5%) were hypoalbuminemic (<3.5 mg/dL). GPS of 0, 1, and 2 were distributed evenly (31.3%, 34.4%, and 34.4%, respectively).

**Table 1:** Baseline clinicopathological features of enrolled patients

**Variables**	
Age (median, range)	63.5 (35–82)
Sex (%)	
Female	5 (15.6)
Male	27 (84.4)
Size of metastasis (cm; median, range)	3.5 (1–10)
Interval: primary diagnosis to adrenalectomy (months; median, range)	8.8 (0–93.8)
Intent to treat (%)	
Palliative	10 (31.3)
Cure	22 (68.8)
Site of primary tumor (%)	
Glottis	1 (3.1)
Esophagus	1 (3.1)
Colon	4 (12.5)
Liver	5 (15.6)
Stomach	3 (9.4)
Kidney	4 (12.5)
Lung	11 (34.4)
Cervix	1 (3.1)
Pancreas	1 (3.1)
Ovary	1 (3.1)
Site of metastasis (%)	
Single	14 (43.8)
Both	5 (15.6)
Adrenal + other site	13 (40.6)
Previous metastasectomy	
No	30 (93.8)
Yes	2 (6.3)
Interval: primary diagnosis to adrenalectomy (%)	
≥6 months (metachronous)	13 (40.6)
<6 months (synchronous)	19 (59.4)
ECOG-PS (%)	
0	5 (15.6)
1	23 (71.9)
2	4 (12.5)
Operative method (%)	
Open	20 (62.5)
Laparoscopic	12 (37.5)
Recurrence after adrenalectomy (%)	
No	12 (37.5)
Yes	20 (62.5)
C-reactive protein (mg/dl,%)	
≤1.0	11 (34.4)
>1	21 (65.2)
Albumin (g/dl,%)	
<3.5	12 (37.5)
≥3.5	20 (62.5)
GPS (%)	
0	10 (31.3)
1	11 (34.4)
2	11 (34.4)

### Surgical treatment and recurrence

The selection of surgical technique (open or laparoscopic) depended on the individual surgeon. Laparoscopic ADx was performed using a retroperitoneal approach. There were no complications or mortalities related to ADx. Recurrence after ADx was slightly high (62.5%).

### Prognostic factors for RFS and OS

The median OS and RFS after ADx in enrolled patients were 10.4 months (range, 0.5-74.3) and 7.3 months (range, 0.5-74.3) respectively. Univariate analyses of the clinicopathological parameters and RFS and OS are shown in Table 2 and Table 3. In the univariate analysis, ADx for cure was significantly associated with a better OS (HR; 0.30 95% CI: 0.10-0.93, p = 0.038). RFS was not associated with intent to treat (for cure, HR; 0.58, 95% CI: 0.23-1.49, p = 0.265). C-reactive protein (>1 mg/dl) showed a worse effect on RFS (HR; 7.55, 95% CI: 1.69-33.6, p = 0.008) and OS (HR; 5.74, 95% CI: 1.24-26.5, p = 0.025). The GPS (=2), together with C-reactive protein and serum albumin level, showed a worse effect on RFS (HR; 6.47, 95% CI: 1.64-25.3, p = 0.007) and OS (HR; 5.33, 95% CI: 1.09-26.0, p = 0.039). Multivariate regression analysis identified the independent negative prognostic factors for OS and RFS (Tables 2 and 3). The independent negative prognostic factors for OS were synchronous metastasis (HR 3.05, 95% CI 1.07-11.94, p = 0.048) and GPS (HR 6.65, 95% CI 1.25-35.23, p = 0.026). The independent negative prognostic factors for RFS were laparoscopic surgery (HR; 4.68, 95% CI: 1.61-13.61, p = 0.005) and GPS (=2, HR; 11.8, 95% CI: 2.50–55.7, p = 0.002).

**Table 2:** Univariate and multivariate analysis of variables affecting RFS

	**RFS (univariate analysis)**		**RFS (multivariate analysis)**	
	**Hazard ratio (95% CI)**	**p-value**	**Hazard ratio (95% CI)**	**p-value**
Age (>63.5 years)	1.84 (0.74-4.63)	0.19		
Sex				
Female	1 (reference)			
Male	0.77 (0.24-2.38)	0.651		
Operative method				
Open	1 (reference)		1 (reference)	
Laparoscopy	1.95 (0.78-4.87)	0.152	4.68 (1.61-13.61)	0.005
Intent to treat				
Palliative	1 (reference)			
Cure	0.58 (0.23-1.49)	0.265		
Synchronous metastasis	1.95 (0.72-5.26)	0.186		
Site of metastasis				
Single	1 (reference)			
Both	0.74 (0.15-3.56)	0.714		
Adrenal + other site	1.02 (0.39-2.60)	0.967		
Site of primary tumor				
Other	1 (reference)			
Liver	0.51 (0.11-2.24)	0.373		
Stomach	0.15 (0.01-1.45)	0.103		
Kidney	0.63 (0.11-3.58)	0.604		
Lung	1.68 (0.57-4.94)	0.346		
Previous metastasectomy				
No	1 (reference)			
Yes	0.33 (0.04-2.55)	0.294		
C-reactive protein (mg/dl)				
≤1.0	1 (reference)			
>1	7.55 (1.69-33.6)	0.008		
Albumin (g/dl)				
≥3.5	1 (reference)			
<3.5	2.03 (0.83-4.93)	0.118		
GPS				
0	1 (reference)		1 (reference)	
1	2.82 (0.73-10.9)	0.132	2.77 (0.61-12.69)	0.189
2	6.47 (1.64-25.3)	0.007	11.80 (2.50-55.70)	0.002
ECOG-PS				
0-1	1 (reference)			
2	2.03 (0.65-6.32)	0.222		

**Table 3:** Univariate and multivariate analysis of variables affecting OS

	**OS (univariate analysis)**		**OS (multivariate analysis)**	
	**Hazard ratio (95% CI)**	**p-value**	**Hazard ratio (95% CI)**	**p-value**
Age (>63.5 years)	1.13 (0.37-3.46)	0.823		
Sex				
Female	1 (reference)			
Male	2.56 (0.33-19.7)	0.367		
Operative method				
Open	1 (reference)			
Laparoscopy	1.81 (0.62-5.29)	0.277		
Intent to treat				
Palliative	1 (reference)			
Cure	0.30 (0.10-0.93)	0.038		
Synchronous metastasis	2.15 (0.68-6.73)	0.187	3.05(1.07-11.94)	0.048
Site of metastasis				
Single	1 (reference)			
Both	0.60 (0.07-5.07)	0.64		
Adrenal + other site	1.19 (0.39-3.61)	0.754		
Site of primary tumor				
Other	1 (reference)			
Liver	0.35 (0.05-2.51)	0.301		
Stomach	0.26 (0.02-2.87)	0.277		
Kidney	0.63 (0.09-4.41)	0.648		
Lung	1.41 (0.34-5.75)	0.626		
Previous metastasectomy				
No	1 (reference)			
Yes	0.04 (0–67.8)	0.397		
C-reactive protein (mg/dl)				
≤1.0	1 (reference)			
>1	5.74 (1.24-26.5)	0.025		
Albumin (g/dl)				
≥3.5	1 (reference)			
<3.5	1.96 (0.68-5.61)	0.2		
GPS				
0	1 (reference)		1 (reference)	
1	3.28 (0.60-17.8)	0.168	2.54 (0.46-14.26)	0.288
2	5.33 (1.09-26.0)	0.039	6.65 (1.25-35.23)	0.026
ECOG-PS				
0-1	1 (reference)			
2	1.81 (0.49-6.62)	0.368		

## Discussion

The adrenal glands are one of the most common sites for metastasis, and the prevalence of isolated adrenal metastasis has increased due to routine surveillance of patients with known malignancy using radiologic examinations based on computed tomography, magnetic resonance imaging, and positron emission tomography [10,12-14]. Although small studies related to adrenalectomy in the setting of metastasis have reported improved survival, patients with adrenal metastasis are frequently regarded as inoperable and have a poor prognosis [7,10,15]. Prognostic factors and surgical indications for adrenalectomy have not been clearly defined, and more series related to these patients in this setting are needed.

Previously, Muth et al. reported the indication for adrenalectomy for adrenal metastasis with a consecutive series of 30 patients, and the independent prognostic factors of favorable survival were adrenalectomy for potential cure, no previous metastasis surgery, and tumor type [9]. Vazquez et al. identified synchronous disease, tumor type, size, burden, and site as risk factors for poor prognosis in univariate analysis [10]. More recently, Howell et al. suggested that prognostic factors included synchronous disease, a short disease-free interval (DFI), and lung primary [16].

In our study, the independent negative prognostic factors for overall survival (OS) were synchronous metastasis, and GPS. Synchronous metastasis was also an independent prognostic factor in our study. With regard to synchronous metastasis, it was explained theoretically that patients with a tumor presenting as a synchronous metastasis growing faster or more aggressively and patients with metachronous disease could be regarded as having more indolent tumors [16]. With regard to the laparoscopic and open approach, the laparoscopic method could be a feasible option even in aggressive tumor because we believe that our study include the more aggressive tumor [17,18]. As mentioned before, synchronous tumor and short disease-free interval are related to more aggressive tumor, and overall survival could reflect the tumor aggressiveness [9,10,16]. Tanvetyanon et al. reported that the median overall survival was shorter (~12 months) for patients with synchronous tumor [8]. In our study, there were 19 synchronous patients (59.4%), and the disease-free interval was 8.8 months. The median overall survival was only 10.5 months. Muth et al. reported nine patients (30%) with synchronous disease, a median DFI of 26 months, and a median survival of 23 months [9]. Howell et al. described 11 synchronous patients (19%), a DFI > 12 months for 39 patients (81%), and an overall median survival of 30 months [16]. Howell et al. preferred open surgery in patients with multi-focal disease and in patients with more aggressive tumor. In our study, more aggressive patients were included, and the laparoscopic approach could be used in those patients in line with results in previous studies. However, additional studies are needed to clarify this point.

In our study, GPS was selected as an independent poor prognostic factor in multivariate analysis. GPS is based on a combination of CRP and albumin and has been evaluated in a variety of cancers, such as renal cancer, breast cancer, non-small cell lung cancer, gastroesophageal cancer, pancreatic cancer, and colorectal cancer [19-24]. CRP is a sensitive marker of systemic inflammation, and elevated CRP concentrations are associated with poorer survival in cancer patients, particularly in patients with advanced disease [25]. The association with elevated CRP levels and a dismal prognosis might reflect the prognostic value of tumor produced interleukin-6, an inducer of CRP production in the liver. Presurgical CRP did correlate significantly with shorter RFS and OS in univariate analysis as like other study. Albumin concentrations reflect both systemic inflammation and the amount of lean tissue [25]. GPS, which is a combination of CRP and albumin levels, reflects the effects of systemic inflammatory response and the process of nutritional decline in advanced cancer [26,27]. However, to our knowledge, there has not been a study to evaluate the prognostic significance in cancer patients with metastatic adrenal lesion. Cancer cachexia and the ECOG performance status have been mentioned as prognostic factors, but ECOG performance status is recognized to be subjective [28,29]. In our study, GPS had a prognostic value superior to that of ECOG-PS. Mcmillan suggested that the GPS is a simple objective measure that can reflect cancer cachexia and predict outcome in patients with cancer [29]. Furthermore, Lamb et al. reported that an elevated GPS prior to surgery might be a useful prognostic indicator in advanced renal cell carcinoma and may alter the decision for surgery [30]. More research will be needed to validate GPS as a risk factor in cancer patients with adrenal metastasis.

Our study has a number of limitations. The study was hampered by selection bias, and several variables are inter-related because of the retrospective study design. Furthermore, the number of patients was relatively small for multivariate analysis, maybe due to this, there were some discrepancies in the results. Hazard ratio (HR) of both adrenal metastasis was lower than that of single metastasis. Moreover, HR of the presence of previous metastasectomy was lower than no history of previous metastasectomy. In addition, heterogenous tumors are included and malignant potential of primary tumor will be the important factor for OS and RFS. However, the number of each tumor type was not sufficient to assess the prognostic value. Larger cohort study also is needed to find out the prognostic value of the malignant potential of primary tumor.

Finally, our results were expressed with respect to the outcomes of RFS and OS, rather than cancer specific survival (CSS). Future studies of prognostic factors should include CSS as an outcome measure.

## Conclusion

In our study, synchronous disease, operation method, and inflammation-based prognostic score were significant prognostic factors for survival associated with adrenalectomy in cancer patients with adrenal metastatic diseases. Synchronous metastasis was also a negative prognostic factor, which is in line with results from a previous study. However, with regard to the operation method, further study is necessary to establish the feasibility of adequate outcomes with a laparoscopic approach in patients with aggressive tumors. GPS appears to be superior to ECOG-PS and could be a simple objective prognostic indicator, but the baseline value of GPS prior to surgery needs to be established in additional studies.

## Competing interests

The authors declare that they have no competing interests.

## Authors’ contributions

SGK participated in the design of the study and performed the statistical analysis. ECH participated in the design of the study, performed the statistical analysis and draft the manuscript. IH, SHK, JGL, SHH, and JEH collected the clinical data. SIJ, TWK, DDK, JJK, and JC made critical revision of the manuscript for important intellectual content. SGK and ECH conceived of the study, and approved the final draft of the manuscript. All authors read and approved the final draft of the manuscript.

## Pre-publication history

The pre-publication history for this paper can be accessed here:

http://www.biomedcentral.com/1471-2490/14/41/prepub

## References

[B1] Bullock WK Hirst AE Jr Metastatic carcinoma of the adrenal Am J Med Sci 1953 226 521 524 10.1097/00000441-195322650-00007 13104403

[B2] Lenert JT Barnett CC Jr Kudelka AP Sellin RV Gagel RF Prieto VG Skibber JM Ross MI Pisters PW Curley SA Evans DB Lee JE Evaluation and surgical resection of adrenal masses in patients with a history of extra-adrenal malignancy Surgery 2001 130 1060 1067 10.1067/msy.2001.118369 11742339

[B3] Lee JE Evans DB Hickey RC Sherman SI Gagel RF Abbruzzese MC Abbruzzese JL Unknown primary cancer presenting as an adrenal mass: frequency and implications for diagnostic evaluation of adrenal incidentalomas Surgery 1998 124 1115 1122 10.1067/msy.1998.92009 9854592

[B4] Kim SH Brennan MF Russo P Burt ME Coit DG The role of surgery in the treatment of clinically isolated adrenal metastasis Cancer 1998 82 389 394 10.1002/(SICI)1097-0142(19980115)82:2<395::AID-CNCR20>3.0.CO;2-T 9445197

[B5] Fong Y Cohen AM Fortner JG Enker WE Turnbull AD Coit DG Marrero AM Prasad M Blumgart LH Brennan MF Liver resection for colorectal metastases J Clin Oncol 1997 15 938 946 9060531 10.1200/JCO.1997.15.3.938

[B6] 1997 Long-term results of lung metastasectomy: prognostic analyses based on 5206 cases. The International Registry of Lung Metastases J Thorac Cardiovasc Surg 1997 113 37 49 10.1016/S0022-5223(97)70397-0 9011700

[B7] Collinson FJ Lam TK Bruijn WM de Wilt JH Lamont M Thompson JF Kefford RF Long-term survival and occasional regression of distant melanoma metastases after adrenal metastasectomy Ann Surg Oncol 2008 15 1741 1749 10.1245/s10434-008-9836-y 18379853

[B8] Tanvetyanon T Robinson LA Schell MJ Strong VE Kapoor R Coit DG Bepler G Outcomes of adrenalectomy for isolated synchronous versus metachronous adrenal metastases in non-small-cell lung cancer: a systematic review and pooled analysis J Clin Oncol 2008 26 1142 1147 10.1200/JCO.2007.14.2091 18309950

[B9] Muth A Persson F Jansson S Johanson V Ahlman H Wängberg B Prognostic factors for survival after surgery for adrenal metastasis Eur J Surg Oncol 2010 36 699 704 10.1016/j.ejso.2010.04.002 20452170

[B10] Vazquez BJ Richards ML Lohse CM Thompson GB Farley DR Grant CS Huebner M Moreno J Adrenalectomy improves outcomes of selected patients with metastatic carcinoma World J Surg 2012 36 1400 1405 10.1007/s00268-012-1506-3 22411083

[B11] Ma X Li H Zhang X Huang Q Wang B Shi T Hu D Ai Q Liu S Gao J Yang Y Dong J Zheng T Modified anatomical retroperitoneoscopic adrenalectomy for adrenal metastatic tumor: technique and survival analysis Surg Endosc 2013 27 992 999 10.1007/s00464-012-2553-4 23239289

[B12] Lo CY van Heerden JA Soreide JA Grant CS Thompson GB Lloyd RV Harmsen WS Adrenalectomy for metastatic disease to the adrenal glands Br J Surg 1996 83 528 531 10.1002/bjs.1800830432 8665251

[B13] Lam KY Lo CY Metastatic tumours of the adrenal glands: a 30-year experience in a teaching hospital Clin Endocrinol (Oxf) 2002 56 95 101 10.1046/j.0300-0664.2001.01435.x 11849252

[B14] Abrams HL Spiro R Goldstein N Metastases in carcinoma; analysis of 1000 autopsied cases Cancer 1950 3 74 85 10.1002/1097-0142(1950)3:1<74::AID-CNCR2820030111>3.0.CO;2-7 15405683

[B15] Kuczyk M Wegener G Jonas U The therapeutic value of adrenalectomy in case of solitary metastatic spread originating from primary renal cell cancer Eur Urol 2005 48 252 257 10.1016/j.eururo.2005.04.004 15936136

[B16] Howell GM Carty SE Armstrong MJ Stang MT McCoy KL Bartlett DL Yip L Outcome and prognostic factors after adrenalectomy for patients with distant adrenal metastasis Ann Surg Oncol 2013 20 3491 3496 10.1245/s10434-013-3050-2 23793361 PMC4879835

[B17] Elashry OM Clayman RV Soble JJ McDougall EM Laparoscopic adrenalectomy for solitary metachronous contralateral adrenal metastasis from renal cell carcinoma J Urol 1997 157 1217 1222 10.1016/S0022-5347(01)64927-9 9120905

[B18] Bonnet S Gaujoux S Leconte M Thillois JM Tissier F Dousset B Laparoscopic adrenalectomy for metachronous metastasis from renal cell carcinoma World J Surg 2008 32 1809 1814 10.1007/s00268-008-9539-3 18330621

[B19] Forrest LM McMillan DC McArdle CS Angerson WJ Dunlop DJ Comparison of an inflammation-based prognostic score (GPS) with performance status (ECOG) in patients receiving platinum-based chemotherapy for inoperable non-small-cell lung cancer Br J Cancer 2004 90 1704 1706 15150622 10.1038/sj.bjc.6601789 PMC2409737

[B20] McMillan DC Crozier JE Canna K Angerson WJ McArdle CS Evaluation of an inflammation-based prognostic score (GPS) in patients undergoing resection for colon and rectal cancer Int J Colorectal Dis 2007 22 881 886 10.1007/s00384-006-0259-6 17245566

[B21] Leitch EF Chakrabarti M Crozier JE McKee RF Anderson JH Horgan PG McMillan DC Comparison of the prognostic value of selected markers of the systemic inflammatory response in patients with colorectal cancer Br J Cancer 2007 97 1266 1270 10.1038/sj.bjc.6604027 17923866 PMC2360467

[B22] Brown DJ Milroy R Preston T McMillan DC The relationship between an inflammation-based prognostic score (Glasgow Prognostic Score) and changes in serum biochemical variables in patients with advanced lung and gastrointestinal cancer J Clin Pathol 2007 60 705 708 10.1136/jcp.2005.033217 16644880 PMC1955069

[B23] Ishizuka M Nagata H Takagi K Horie T Kubota K Inflammation-based prognostic score is a novel predictor of postoperative outcome in patients with colorectal cancer Ann Surg 2007 246 1047 1051 10.1097/SLA.0b013e3181454171 18043109

[B24] Read JA Choy ST Beale PJ Clarke SJ Evaluation of nutritional and inflammatory status of advanced colorectal cancer patients and its correlation with survival Nutr Cancer 2006 55 78 85 10.1207/s15327914nc5501_10 16965244

[B25] McMillan DC An inflammation-based prognostic score and its role in the nutrition-based management of patients with cancer Proc Nutr Soc 2008 67 257 262 10.1017/S0029665108007131 18452641

[B26] Forrest LM McMillan DC McArdle CS Angerson WJ Dunlop DJ Evaluation of cumulative prognostic scores based on the systemic inflammatory response in patients with inoperable non-small-cell lung cancer Br J Cancer 2003 89 1028 1030 10.1038/sj.bjc.6601242 12966420 PMC2376960

[B27] Roxburgh CS McMillan DC Role of systemic inflammatory response in predicting survival in patients with primary operable cancer Future Oncol 2010 6 149 163 10.2217/fon.09.136 20021215

[B28] Fearon KC Voss AC Hustead DS Cancer Cachexia Study Group Definition of cancer cachexia: effect of weight loss, reduced food intake, and systemic inflammation on functional status and prognosis Am J Clin Nutr 2006 83 1345 1350 16762946 10.1093/ajcn/83.6.1345

[B29] McMillan DC Systemic inflammation, nutritional status and survival in patients with cancer Curr Opin Clin Nutr Metab Care 2009 12 223 226 10.1097/MCO.0b013e32832a7902 19318937

[B30] Lamb GW Aitchison M Ramsey S Housley SL McMillan DC Clinical utility of the Glasgow Prognostic Score in patients undergoing curative nephrectomy for renal clear cell cancer: basis of new prognostic scoring systems Br J Cancer 2012 106 279 283 10.1038/bjc.2011.556 22166802 PMC3261680

